# Deregulated expression of hnRNP A/B proteins in human non-small cell lung cancer: parallel assessment of protein and mRNA levels in paired tumour/non-tumour tissues

**DOI:** 10.1186/1471-2407-10-434

**Published:** 2010-08-17

**Authors:** Georgios Boukakis, Meropi Patrinou-Georgoula, Maria Lekarakou, Christos Valavanis, Apostolia Guialis

**Affiliations:** 1 RNA Processing Program, Institute of Biological Research and Biotechnology, National Hellenic Research Foundation, 48 Vas Constantinou Avenue, 11635 Athens, Greece; 2 Molecular Pathology Unit, Dept of Pathology, Metaxa Cancer Hospital, Piraeus, Greece

## Abstract

**Background:**

Heterogeneous nuclear ribonucleoproteins (hnRNPs) of the A/B type (hnRNP A1, A2/B1, A3) are highly related multifunctional proteins participating in alternative splicing by antagonising other splicing factors, notably ASF/SF2. The altered expression pattern of hnRNP A2/B1 and/or splicing variant B1 alone in human lung cancer and their potential to serve as molecular markers for early diagnosis remain issues of intense investigation. The main objective of the present study was to use paired tumour/non-tumour biopsies from patients with non-small cell lung cancer (NSCLC) to investigate the expression profiles of hnRNP A1, A2/B1 and A3 in conjunction with ASF/SF2.

**Methods:**

We combined western blotting of tissue homogenates with immunohistochemical examination of fixed tissue sections and quantification of mRNA expression levels in tumour versus adjacent normal-looking areas of the lung in the same patient.

**Results:**

Our study, in addition to clear evidence of mostly uncoupled deregulation of hnRNPs A/B, has revealed hnRNP A1 to be the most deregulated protein with a high frequency of over-expression (76%), followed by A3 (52%) and A2/B1 (43%). Moreover, direct comparison of protein/mRNA levels showed a lack of correlation in the case of hnRNP A1 (as well as of ASF/SF2), but not of A2/B1, suggesting that different mechanisms underlie their deregulation.

**Conclusion:**

Our results provide strong evidence for the up-regulation of hnRNP A/B in NSCLC, and they support the existence of distinct mechanisms responsible for their deregulated expression.

## Background

The biogenesis of mRNA in higher eukaryotes is largely based on the interplay of a large number of RNA-binding proteins (RBPs) [1]. Heterogeneous nuclear ribonucleoproteins (hnRNPs) are RBPs that are essential players in mRNA metabolism, acting as coordinators of post-transcriptional events (splicing, transport, cellular localisation, decay and translation of mRNA) by participating in an extensive network of RNA-RBP interactions. Individual hnRNPs also function in several other cellular processes, like transcription, DNA repair, telomere biogenesis and cell signalling (reviewed in [2-4]). As a consequence of their multiple roles in the regulation of gene expression, any malfunctioning, especially with respect to their deregulated expression in cancer, is expected to affect the physiological network of RNA-RBP interactions [5,6].

More than 20 distinct hnRNP proteins have been identified in human cells, designated hnRNPs A1 to U in increasing molecular size from 32 to 110 kDa. They represent a family of abundant nuclear proteins, many of them sharing common structural motifs, exhibiting multiple isoforms (products of alternative splicing, as well as of post-translational modification) and having the ability to shuttle between the nuclear and cytoplasmic compartments [7,8]. The hnRNP A/B group includes members of 32-40 kDa having in common two tandem N-terminal RNA-binding domains of the RRM/RBD type and a C-terminal auxiliary domain rich in glycine (2xRBD-gly). The most abundant and best characterised are hnRNP A1 and A2/B1, as well as the recently identified hnRNP A3, all three of which share a high degree of sequence homology and the presence of several isoforms originating mainly from alternative splicing (reviewed in [4]). In particular, hnRNP A2/B1 refers to two isoforms; the major hnRNP A2 and the minor B1 form that results from the inclusion of an extra exon of 12 amino acid residues [9]. Their proportions, both in protein and mRNA levels, vary in different cells and tissues, with B1 constituting roughly 2-5% of A2 [10,11]. The major nuclear function of hnRNPs is thought to be in splicing and particularly in alternative splicing. This is especially the case for hnRNP A1 and A2/B1, which have been shown to antagonise, in a concentration dependent manner, protein members of the SR group, notably ASF/SF2, and to influence the mode of splicing of mRNA target molecules [12]. Changes in the expression levels of hnRNP A/B and ASF/SF2 have also been reported in human colon adenocarcinomas [13] and in a mouse model of lung carcinogenesis [14].

The overall expression of hnRNP A/B proteins is known to be tightly regulated during development and to be tissue- and cell-type specific [11,15]. In rodent and human lung tissues, high hnRNP A/B levels occur during embryonic development that drop dramatically in the adult lung [16]. Moreover, recent studies that have simultaneously reduced the protein levels of both hnRNP A1 and A2 in cultured cells have demonstrated a role for them in cell proliferation [4], as well as a strong association of their reduction with cell death in cancer but not in non-cancer immortalised cells, indicating that cancer cells require these proteins for viability [17]. In general, the aberrant expression of hnRNPs in cancer cells has been documented by a series of studies [5,18,19] and found to be associated with alterations in their protein levels (usually over-expression, but also down-regulation), mRNA abundance, cellular localisation, isoform types and post-translational modifications (like patterns of phosphorylation). Of particular importance are initial findings linking over-expression of hnRNP A2/B1 with early stages of human lung carcinogenesis. In an archival study using exfoliated epithelial cells in sputum, hnRNP A2/B1 over-expression was considered to be a promising new diagnostic marker able to accurately predict lung carcinogenesis at least a year before the appearance of any cytological findings [19,20]. Correlations between hnRNP A2/B1 over-expression, microsatellite alterations and loss of heterogeneity in early stages of malignancy have been reported [21,22]. Because of the pressing need for early diagnostic and prognostic biomarkers, these initial results provided the impetus for a number of subsequent studies. Most reports have focused on the expression patterns of hnRNP A2/B1 and/or the splicing isoform B1 alone [23-29], whereas one study included additional hnRNPs (hnRNP A1, C1/C2, K) [30]. hnRNP A2/B1 over-expression is not unique to lung cancer, as it has been also recorded in squamous cell carcinomas of oral and esophageal cancers [25], pancreatic cancer [31] and in cell lines established from human liver, colon and stomach cancers [23].

To date, because of contradictory findings, no consensus exists as to the significance of hnRNP A2/B1 over-expression in early lung carcinogenesis. Moreover, the exact role of hnRNP A/B proteins in carcinogenesis and the mechanism of their deregulation remain unclear. To further elucidate the roles of hnRNP A/B (A1, A2/B1, A3) in conjunction with the splicing factor ASF/SF2 in lung cancer, we present here an evaluation of their protein and mRNA levels in paired tumour/non-tumour biopsies from NSCLC patients. Our findings should help to unravel the mode and consequences of their deregulated expression in lung carcinogenesis.

## Methods

### Tissue samples and patients

Fresh lung biopsies (kept in liquid nitrogen) from a total of 21 patients who had been operated on for non-small cell lung cancer (NSCLC), together with adjacent normal-appearing lung tissue, were randomly selected from the tissue banks of the Department of Pathology, Metaxa Cancer Hospital, Piraeus, Greece. The material was obtained by lung lobe resection and included the following tumour subtypes: six adenocarcinomas, one bronchioalveolar carcinoma, three large cell carcinomas, nine squamous cell carcinomas and two adenosquamous carcinomas. The ages of the patients ranged from 54 to 78 (average 66) with 18 males and 3 females. Additional clinico-pathological data refer to the differentiation status of the tumour, the state of histiocytes and pleural invasion, the presence of metastasis in lymph nodes (LNs) and the tumour-to-node metastasis (TNM) index. The study was performed under the approval of the Metaxa Cancer Hospital Bioethics committee of Greece.

### Antibodies

The following mouse monoclonal antibodies were obtained from Santa Cruz Biotec and were used at the indicated dilutions in western blotting: anti-hnRNP A1, 4B10 (1:1,000); anti-hnRNP A2/B1, DP3B3 (1:1,000); anti-hnRNP K/J, 3C2 (1:500); anti-hnRNP SF2/ASF, 33652 (1:500). Anti-β-actin mouse monoclonal MAB1501 antibody was purchased from Chemicon (1:4,000) and rabbit polyclonal anti-hnRNP A3 (50949) antibody was from Abcam Co. (1:2,000). The secondary antibodies were horseradish peroxidase-conjugated anti-mouse (Sigma Co) and anti-rabbit (Chemicon-Milipore) IgG.

### Tissue homogenisation and western blotting

For every patient, a small portion of the biopsy, taken from either the cancer area or a remote (> 5 cm) non-involved lung tissue site, was immediately lysed in a protein lysis buffer (9.5 M urea, 2% NP-40, 5% β-mercaptoethanol). Complete homogenisation of the tissue was done with a rotor-stator homogeniser TissueRuptor (Qiagen) and TCA precipitation of total protein followed. The protein pellet was resuspended in a small volume (50-100 μl) of SDS sample buffer and roughly similar amounts of protein (estimated by Coomassie blue staining of a test gel) of paired tumour and non-tumour samples were resolved by SDS-PAGE and transferred to a nitrocellulose membrane. After incubation, the membrane was incubated in blocking buffer (10 mM Tris-HCl pH 7.5, 0.1 M MgCl_2_, 5% low-fat skim milk, 0.5% Τween-20, 0.1% Τriton-X100) with gentle rocking overnight at 4°C using the appropriate dilution of the antibody in blocking buffer. The antigen-antibody reaction was visualised following incubation with an HRP-conjugated secondary antibody and application of the ECL detection system (Amersham Biosciences, UK). Semi-quantitative estimation of the test protein was done by scanning the appropriate band on X-ray film in a digital scanner with Image Quant software (Molecular Dynamics, CA) in parallel with that of β-actin run within the same sample, which was used as an internal loading control for normalisation. In the relative quantitation of western blots, care was taken to compare roughly similar detection signals that were within the linearity range of the X-ray film scanner.

### Immunohistochemistry

Pairs of formalin-fixed, paraffin-embedded sections corresponding to a resected NSCLC biopsy and its neighbouring normal-looking area were prepared. Fixed sections from 18 out of the 21 patients that were included in the western blotting analysis were used for immunohistochemical detection of hnRNP A1 and A2/B1 proteins using the above-described anti-hnRNP A1 and anti-hnRNP A2/B1 monoclonal antibodies at 1:300 dilutions. Sections were deparaffinised and incubated with the antibody overnight at 4°C. Following incubation with anti-mouse secondary IgG, immunostaining was developed with diaminobenzidine and counterstained with hematoxylin. Interpretation of the staining records and assignment of scoring indexes was done by two independent pathologists.

### Real-time PCR analysis (RT-qPCR)

Total RNA was extracted from a small portion of the biopsies with the RNeasy Mini Kit (Qiagen) according to the manufacturer's protocol. On-column DNA digestion (RNase-Free DNase Set, Qiagen) was used to ensure the absence of DNA from the samples. The quality of the isolated RNA was tested by its optical density (260/280 over 1.5) and the presence of 28S and 18S ribosomal RNA resolved by agarose gel electrophoresis. The expression of hnRNP A1, A2/B1, B1 and ASF/SF2 was quantified by real-time PCR (RT-qPCR). Total RNA (1 μg) was reverse-transcribed using Superscript II (Invitrogen) reverse transcriptase and oligo-dT primers according to the manufacturer's instructions. Real-time PCR reaction products were synthesised and quantified by SYBR Green I (iQ SYBR Green Supermix, Biorad) on an iQ5 Real-Time PCR Detection System (Biorad) using the ribosomal LP32 gene as an internal control for normalisation. All assays were performed in triplicate in a 25-μl two-step reaction. The specificity of the amplified PCR products was assessed by melting curve analysis and agarose gel electrophoresis of a small aliquot of the reaction followed by staining with ethidium bromide. Relative mRNA levels of test proteins were determined by the Normalised Gene Expression method using iQ5 Optical System Software. The efficiency of the qPCR reaction was measured in separate assays using serial dilutions of cDNA obtained from total RNA from the A549 lung cell line for every gene of interest.

The specific primers were:

hnRNP A1: Forward 5'-CCAGAGAAGATTCTCAAAGACC-3';

Reverse 5'-CTTCAGTGTCTTCTTTAATGCC-3'

hnRNP A2/B1: Forward 5'-AGCTTTGAAACCACAGAAGAA-3';

Reverse 5'-TTGATCTTTTGCTTGCAGGA-3'

hnRNP B1: Forward 5'-TGTTCCTTTGGAGAGGAAAAAG-3';

Reverse 5'-TTGATCTTTTGCTTGCAGGA-3'

ASF/SF2: Forward 5'-CTTGGTGGGAAGGCCTGTT-3';

Reverse 5'-AGATGCGGCAATCGTTGTTC-3'

RPL32: Forward 5'-TTAAGCGTAACTGGCGGAAAC-3'

Reverse 5'-GAGCGATCTCGGCACAGTAA-3'

### Statistical analysis

The non-parametric Wilcoxon ranked-sum test was used to statistically analyse the protein and mRNA levels (from western blotting and RT-qPCR analyses, respectively) of the paired tumour and non-tumour tissues. Values of *p *< 0.05 were considered to be significant.

## Results

### hnRNP protein levels in paired tumour/non-tumour lung tissue specimens from patients with NSCLC

This study included 21 patients who had been operated on for non-small cell lung cancer (NSCLC). Table 1 presents the list of patients along with the available clinico-pathological data. For every patient, tumour subtype, differentiation stage (equal numbers of either low or intermediate stage), extent of histiocyte infiltration, and presence of pleura invasion is noted. In addition, metastases in lymph nodes (LNs) as well as the tumour-to-node metastasis (TNM) index with the application of the new 7^th ^version of TNM classification of the lung cancer are indicated. The majority of tumours were associated with a low to medium differentiation stage and absence of metastasis (M0).

**Table 1:** Clinicopathological characteristics of the 21 NSCLC patients (ID # M1-M27)

A/A	SEX	AGE	TUMOR TYPE	DIFFERE-NTIATION	HISTIOCYTES	PLEURAL INVASION	LNs	TNM
M1	M	66	Adeno	Low	FEW	NO	NO	T2aN0M0
M2	M	57	Adeno/Bronch	Medium	YES	NO	NO	T3N0M0
M7	M	54	SqCC	Medium	YES	NO	1+	T1bN1M0
M9	M	63	SqCC	Medium	FEW	YES *	1+	T1bN1M0
M10	M	57	SqCC	Medium/Low	FEW	YES *	NO	T2bN0M0
M12	M	65	LCC	Low	FEW	NO	YES	T3N2M0
M13	M	67	SqCC	Medium/Low	YES	NO	NO	T1bN0M0
M14	M	69	LCC	Low	FEW	YES *	NO	T2bN0M0
M15	F	55	Adeno/SqCC	Low	FEW	YES *	YES	T2aN1M0
M16	F	76	Adeno	Medium	FEW	YES *	YES	T3N1M0
M17	M	60	Adeno	Medium	FEW	NO	NO	T3N0M0
M18	M	75	LCC/Adeno	Low	NO	NO	1+	T2aN1M0
M19	F	55	SqCC	Medium/Low	NO	YES pleural nodules/bone invasion	1+	T4N1M1b
M20	M	74	Adeno/SqCC	Low	NO	NO	NO	T2aN0M0
M21	M	70	Adeno	Low	NO	YES *	NO	T1bN0M0
M22	M	71	Adeno	Low	YES	NO	1+	T2aN3M0
M23	M	78	SqCC	Low	YES	N/A	N/A	N/A
M24	M	72	SqCC	Medium	NO	YES *	YES	T2aN2M0
M25	M	60	Adeno	Low	N/A	N/A	N/A	N/A
M26	M	62	SqCC	Low	N/A	N/A	N/A	N/A
M27	M	58	SqCC	Low	N/A	N/A	N/A	N/A

SqCC: Squamous cell carcinoma; Adeno: Adenocarcinoma; LCC: Large cell carcinoma; Bronch: Bronchioalveolar; LNs: Lymph nodes; TNM: Tumour-to-node metastasis (7^th ^TNM classification version); M0: absence of metastasis. *pleural invasion with no pleural penetration/no pleural nodules.

For each of the 21 patients, paired biopsies from the tumour and from an adjacent normal-appearing area were provided. Total homogenate was prepared from a portion of the frozen tissue, followed by protein resolution by SDS-PAGE. Western blotting was then used to immunodetect selected members of the hnRNP protein family. The parallel immunodetection of β-actin, serving as a cellular protein index, was used to normalise for protein loading. Densitometric scanning of the identified antigenic bands on the blots was used to obtain semi-quantitative estimates of the hnRNP protein levels relative to β-actin within the same sample. We focused our study on the major hnRNP A/B type proteins that include, in addition to the hnRNP A2/B1 currently implicated mostly in lung cancer, the hnRNP A1 and A3 protein species. In addition to hnRNP A/B, we also looked at the relative levels of hnRNP K (a protein commonly altered in several types of human cancer [6,18]) using an antibody targeting the pair of hnRNP K/J proteins. We note that the assessment of hnRNP K protein level was limited to 16 (out of 21) patients due to inefficient amount of tissue available.

**Figure 1:**
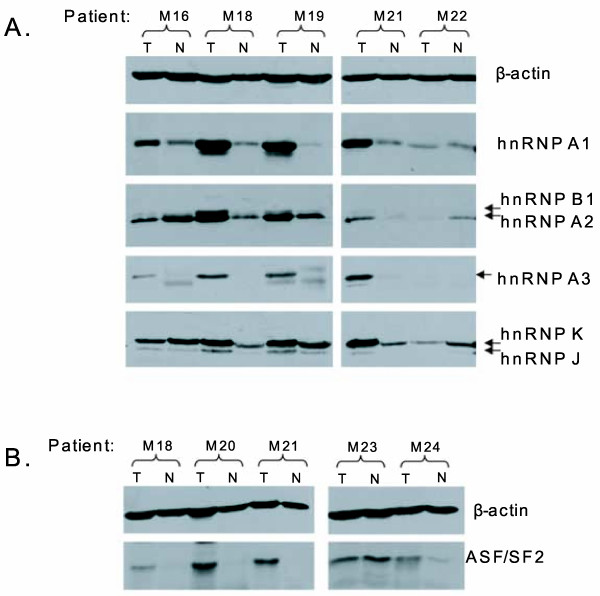
**A representative western blotting analysis of lung tissue homogenates**. Total protein obtained from paired tumour (T) and non-tumour (N) tissues of NSCLC patients was resolved by SDS-PAGE (10% gel) followed by transfer to nitrocellulose membrane and western blotting. **(A) **Immunodetection within the same material of the hnRNP proteins (hnRNP A1, A2/B1, A3 and K/J) and **(B) **of the ASF/SF2 splicing factor was done by incubating the membrane with the appropriate antibodies. The parallel detection of β-actin served as an internal loading control for estimating relative protein expression levels.

Α representative western blot for the immunodetection of β-actin, hnRNP A/B (A1, A2/B1, A3) and K/J proteins is shown in Figure 1A, which presents 5 out of the 21 tumour/non-tumour pairs analysed. Western blotting analysis clearly showed a broad range of altered hnRNP protein expression levels between the paired tumour (T) and non-tumour (N) tissues. After normalising to β-actin, the relative expression level of each of the hnRNP proteins was calculated. For hnRNP A2/B1, the relative amount refers to both the A2 and B1 isoforms that are recognised by the anti-A2/B1 DP3B3 monoclonal antibody. Although in several cases B1 was clearly visible on the gel, it was not sufficiently separated from A2 to allow individual estimates. This was also the case for the homologous hnRNP K and J protein species that were considered together. The differences in the hnRNP expression levels between the paired specimens are depicted as relative T/N fold-changes for all the patients included in this study. A relative fold-change of 1 indicates cases with unaltered levels. Cases with clear up-regulation, cases without an appreciable change and cases with down-regulation were all observed.

**Figure 2:**
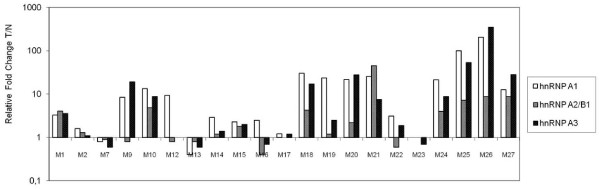
**Comparison of the relative expression levels of the hnRNP A/B proteins in paired tumour and normal biopsies**. A histogram presenting the relative fold change of the tumour-to-normal (T/N) value of paired biopsies for hnRNP A1, A2/B1 and A3 protein levels individually for every patient (M1-M27) included in this analysis.

Table 2 presents the cumulative data on the relative T/N fold-change observed for each hnRNP protein member with respect to the range of T/N ratio and frequency of over-expression for all the patients examined. Note that in the presentation of the T/N range, we have excluded two patients (M25, M26) because the relative level of hnRNP A/B in the normal tissue was very low (almost at a background level) compared to the tumour tissue, which led to a high T/N fold-change (over 100). To apply rather stringent criteria on protein over-expression that was based on semi-quantitative estimates, we deliberately raised the baseline of the relative T/N fold-change from 1 (non-altered levels) to 2. As seen, a broad range of relative T/N fold-change was observed for each protein tested, with hnRNP A1 having the broadest range, followed by hnRNP A3. Similarly, the recorded frequency of over-expression was higher for hnRNP A1 (76%), followed by A3 (52%), A2/B1 (43%) and K/J (38%). The most statistically significant values of over-expression were obtained for A1, followed by A3. Less significant were the estimates of A2/B1, and even less were those for K/J. Cases with a high degree of over-expression (T/N > 10) were also included, and these were found to be higher for hnRNP A1 (43%), followed by A3, A2/B1 and K/J (see Figure 2). The above findings taken together clearly indicate that hnRNP A1 is the most highly and frequently deregulated protein in NSCLC.

**Table 2:** Protein-level estimates: Western blotting results

Fold-change T/N (Protein-level)			
**Protein**	**Range of T/N**	**Frequency of over-expression T/N > 2 *(no. of cases)***	**Significance (*p*-value)**

hnRNP A1	0.4 - 30.0	76% *(16/21)*	0.0006
hnRNP A2/B1	0.4 - 9.0	43% *(9/21)*	0.0199
hnRNP A3	0.6 - 28.0	52% *(11/21)*	0.0025
hnRNP K/J	0.2 - 7.0	38% *(6/16)*	0.0319
ASF-SF2	0.1 - 19.0	31% *(5/16)*	0.4228

Based on the histogram shown in Figure 2, a direct comparison was made individually for every patient included in this study of the three homologous hnRNP A/B type proteins (hnRNP A1, A2/B1 and A3) with respect to both their frequency and the degree of their altered expression. Overall, the cases with up-regulated hnRNP A/B levels were more common than those with down-regulated hnRNP A/B levels. We note, however, that in roughly 20% of the patients with up-regulated levels, there was down-regulation in at least one of the hnRNP A/B proteins. This comparison provides clear evidence that deregulation among the hnRNP A/B proteins is largely uncoupled, as exemplified by their different degree of over-expression and (mainly) by the cases with opposite (up versus down) deregulation in the same patient. With respect to the relative T/N fold-changes in hnRNP K/J, there was mostly parallel expression with hnRNP A1 (data not shown).

As stated in the Background section [12-14], the antagonistic behaviour between hnRNP A/B proteins and the splicing factor ASF/SF2 in the splicing of target mRNA molecules and the existence of a strong link between deregulated splicing events and cancer are important issues to consider. We therefore extended the western blotting analysis of hnRNPs to include the immunodetection of ASF/SF2 protein in the same sample. This analysis included only 16 (out of 21) patients for the reasons stated in the case of hnRNP K. In Figure 1B, a representative blot of five biopsies is shown for ASF/SF2 in parallel with β-actin as the loading control. Similarly to the hnRNPs, we compared the relative expression levels of ASF/SF2 in the tumour (T) and non-tumour (N) specimens and obtained estimates of the relative T/N fold-change in the same patient. As presented in Table 2, compared to the hnRNP proteins, the frequency of ASF/SF2 over-expression was low (31% with T/N > 2), and moreover, it was correlated with a very poor significance value (*p *= 0.42). In the total number of biopsies (n = 16) included in this analysis, and in direct comparison with hnRNP A1 as the protein with the highest frequency and degree of altered expression in the tumours, a high frequency (75%) of concurrent deregulation of ASF/SF2 and hnRNP A1 was apparent, representing mainly over-expression of both proteins (data not shown). However, there were fewer cases with ASF/SF2-relative T/N fold-changes over 10 (12%; 2/16) than with hnRNP A1 over 10 (43%; 9/16).

### Evaluation of the expression pattern of hnRNP A1 and A2/B1 by immunohistochemistry

To investigate the extent to which the western blotting results were consistent with immunohistochemistry results, the anti-hnRNP A1 and A2/B1 antibodies were also used for *in situ *visualisation of the respective antigenic proteins on formalin-fixed paraffin-embedded tissue sections prepared from the same group of patients. Specific cellular staining, both nuclear and cytoplasmic, was recorded and compared between the tumour and the surrounding normal-appearing area of the biopsy from each patient. The degree of immunostaining was scored 0 for no staining, 1 for low staining, 2 for intermediate staining and 3 for high staining. In line with other relevant immunohistochemical studies [29,30], we also noted a variable number of cells with no expression at all (0) next to stained cells within the same area. There were also some cases with extensive heterogeneity in the staining distribution of epithelial cells within the non-tumour area (a score of 1, 2 or even 3), possibly related to the clonality of pre-cancerous cells [32].

**Figure 3:**
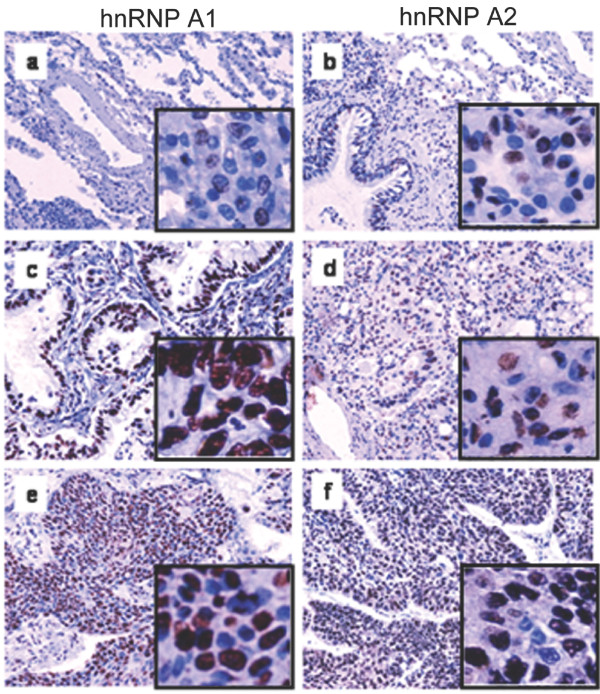
**Estimates of hnRNP A1 and A2/B1 expression levels by immunohistochemistry**. A representative picture showing staining with anti-hnRNP A1 or anti-hnRNP A2/B1 monoclonal antibodies of fixed lung sections of either non-involved (normal; **a **and **b**) or lung cancer tissues of the adenocarcinoma (**c **and **d**) or squamous cell carcinoma (**e **and **f**) subtype. Original magnification: all x200. A representative tissue area corresponding to panels a - f was selected and is shown in a magnified view as an insert picture.

A representative picture is shown in Figure 3 of the hnRNP A1 or A2/B1 antibody staining of selected non-involved (normal; panels a and b) and cancer tissues of an NSCLC type adenocarcinoma (panels c and d) and an NSCLC type squamous cell carcinoma (panels e and f). In the great majority of cases (see the selected case in Figure 3c and 3e), hnRNP A1 was recorded in cells of the tumour site with an intermediate to high range of nuclear staining (score 2 to 3) and practically undetected cytoplasmic staining; in addition, there were some cells with no expression at all. This contrasted with the picture of the normal site showing low level or total lack of nuclear staining (score 1 or 0) (see Figure 3a). However, a few exceptional biopsies presenting broader heterogeneity (score 0, 1, 2 and low numbers of 3) should be noted. Overall, good agreement with the western blotting results was found. In particular, all paired biopsies with a relative T/N fold-change over 10 (43%) were scored with a high (3) nuclear staining in the tumour site. Unlike that of hnRNP A1, the immunostaining pattern of hnRNP A2/B1 was not as distinct, mainly due to cells in the non-tumour site of the biopsy having a broad spectrum of nuclear staining (several with a score of 2 and 3) when contrasted with the corresponding low levels of hnRNP A1 (Figure 3b and 3a, respectively). In the tumour site, all biopsies had a high (3) score for nuclear hnRNP A2/B1 and (with the exception of one) low cytoplasmic hnRNP A2/B1 staining (see examples in Figure 3d and 3f). After taking into consideration this apparently high baseline level of hnRNP A2/B1 in the normal-appearing site of the tumour, there was good correspondence with the relative expression levels of the protein; 43% over-expression as estimated by western blotting. This was highlighted by the high frequency (over 50%) of tumour samples without up-regulated levels of hnRNP A2/B1 (relative T/N fold-change less than 2), unlike A1 (Figure 2). Lastly, our immunohistochemical study did not provide data that correlated with either the sub-type (squamous vs. adenocarcinoma) or the differentiation (low vs. intermediate) stage of the tumour.

### Direct comparison of protein and mRNA levels of hnRNP A/B and ASF/SF2 in paired tumour/non-tumour tissues

A major goal of our study was to investigate cancer-related changes in the protein and mRNA levels of both hnRNPs and ASF/SF2 in the same biopsy. To this end, in parallel with protein isolation, a small portion of the biopsy was used to obtain total RNA. Paired tumour/non-tumour biopsies were used to obtain total RNA, and an equal amount was subsequently reverse transcribed and subjected to real-time PCR amplification (RT-qPCR) using primers specific for hnRNP A1, A2/B1, the B1 isoform alone and ASF/SF2. As with the protein estimation, the mRNA level was reported as the relative fold-change between the paired tumour and non-tumour (T/N) sites for each patient. For mRNA estimates, we used a lower baseline setting (T/N fold-change 1.5, instead of 2 in the case of the protein estimates) taking into account the use of the quantitative RT-qPCR method.

Table 3 presents the cumulative data on the range and frequency of mRNA over-expression, based on estimates provided by a number of 15 to 20 patients (as indicated) for whom sufficient amount of extracted RNA was available. With the baseline of T/N fold-change at 1.5, the frequency of mRNA up-regulation was 15% for hnRNP A1, 40% for A2 and B1 together and 37% for B1 alone. A frequency of 20% was estimated in the case of ASF/SF2 mRNA. Most importantly, a statistically significant value of mRNA over-expression was seen only for A2/B1 mRNA, whereas the least significant value was for A1.

**Table 3:** mRNA-level estimates: Real-time PCR results

Fold-change T/N (mRNA level)			
**mRNA**	**Range of T/N**	**Frequency of over-expression T/N > 1.5 *(no. of cases)***	**Significance (*p*-value)**

hnRNP A1	0.2 - 5.0	15% *(3/20)*	0.6407
hnRNP A2/B1	0.4 - 30.0	40% *(8/20)*	0.0196
hnRNP B1	0.4 - 14.4	37% *(7/19)*	0.1650
ASF-SF2	0.4 - 2.0	20% *(3/15)*	0.1182

**Figure 4:**
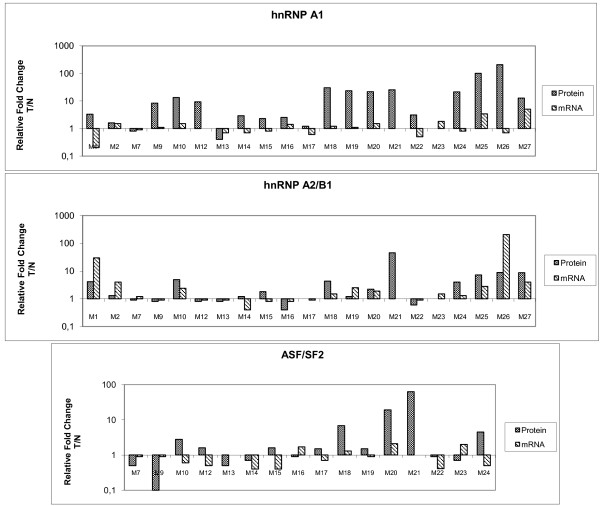
**Direct comparison of hnRNP A/B and ASF/SF2 protein and mRNA levels in paired tumour and normal biopsies**. Histograms presenting the relative fold change in the tumour to normal (T/N) value of paired biopsies for hnRNP A1 (upper panel), A2/B1 (middle panel) and ASF/SF2 (lower panel) protein and mRNA levels in the indicated patients.

From the comparison of the range and frequency of protein over-expression (Table 2) and the mRNA-level estimates shown in Table 3, it became obvious that in the case of hnRNP A1 there was a lack of correspondence between its protein and mRNA levels. This is also clearly shown in the histogram of Figure 4 (panel hnRNP A1), which directly compares protein and mRNA levels for every patient included in this analysis. The high frequency of hnRNP A1 over-expression (76%) was not in line with the corresponding 15% value of its mRNA levels. In fact, in 33% of the cases, there was opposing regulation, with up-regulated levels of the protein and concurrent down-regulated mRNA. A mismatch in the range of altered expression was also apparent because in almost one-third of cases there was a much greater degree of protein over-expression compared to mRNA. In the case of hnRNP A2/B1, this mismatch was not apparent (Figure 4, panel hnRNP A2/B1)), as the same frequency of over-expression (40%) was estimated for both protein and mRNA levels. There were also cases of opposing regulation of A2/B1 protein/mRNA levels at a frequency of 14% (compared to 33% for A1). In clear contrast to hnRNP A1, there were several cases (4/21) that presented a higher fold-change in the mRNA level compared to the protein. With respect to the B1 isoform alone, the change in its mRNA level was within the range (about 40%) recorded for the sum of A2 and B1 (see Tables 2 and 3). However, due to the lack of direct estimates of hnRNP B1 protein levels, a comparison between its protein and mRNA levels could not be made.

The RT-qPCR analysis of the ASF/SF2 splicing factor showed a 20% frequency of mRNA up-regulation, an estimate that was close to the 15% of hnRNP A1. The data presented in the histogram of Figure 4 (panel ASF/SF2), where the direct comparison of ASF/SF2 protein and mRNA level is depicted, reveal a high occurrence (over 50%) of opposing changes. With the exception of one biopsy, this involved over-expression of the protein and concurrent down-regulation of the mRNA, a situation resembling that of hnRNP A1. In addition, a strict comparison of the mRNA levels of ASF/SF2 and hnRNP A1 revealed that 10 out of 15 biopsies exhibited parallel changes (either up- or down regulation). Thus, similarly to the changes at the protein level, the cancer-related alterations of ASF/SF2 mRNA were in line with those of hnRNP A1.

## Discussion

The main advantage of the present study was the use of paired tumour/non-tumour biopsies from NSCLC patients for estimating both protein and corresponding mRNA levels of the hnRNP protein species under investigation. The study was based on the combined application of western blotting and immunohistochemistry for semi-quantitative estimates of protein levels and RT-qPCR analysis for the quantification of steady-state mRNA levels.

A series of studies have reported the altered expression of hnRNPs, mainly of hnRNP A2/B1, in lung carcinogenesis. Most of the studies used immunohistochemistry on resected NSCLC tissues or immunocytochemistry on lung cancer cell lines [25-29,33,34]. In several cases, western blotting, northern blotting and/or RT-PCR were also included [20,22-24,30,35]. So far, our study is unique with respect to the exclusive application of paired tumour/non-tumour lung biopsies and direct comparison of protein and mRNA levels of particular hnRNP protein species. It is believed that biopsies are better suited for the study of lung carcinogenesis than cultured human cell lines (either established or primary cultures), which exhibit altered growth rates. In general, it has been difficult to have proper normal control cell lines because immortalised non-cancerous cells have hnRNP levels comparable to those of cancerous cells. This needs to be taken into consideration because expression levels of hnRNPs are tightly linked to cell proliferation [4].

Initial and subsequent reports in the literature have pointed to the altered expression pattern of hnRNP A2/B1 protein and mRNA in lung cancer. This was taken as a feature of cellular neoplastic transformation preceding morphological differentiation, and it could be useful for the early detection of lung cancer [20,33,34,36]. A high frequency of increased expression of hnRNP A2/B1, as well as of other hnRNPs (A1, C1/C2, K), was found by immunostaining human SCLC and NSCLC cell lines as well as NSCLC biopsies [30]. In the cell lines, the average levels of hnRNP A2/B1 and A1 mRNA were higher in SCLC than in NSCLC, as was the ratio of A2 to B1 mRNA (average value 7.2 and 6.3 in SCLC and NSCLC, respectively) [30]. However, the initial encouraging findings on hnRNP A2/B1 over-expression have come into question with respect to its usefulness as a diagnostic biomarker for early lung cancer. This is because other studies claimed that instead, the B1 splicing isoform was more significantly elevated both at the protein and mRNA level in lung cancer. The application of polyclonal antibodies exclusively recognising the hnRNP B1 variant resulted in strong nuclear staining, mainly of squamous cell carcinoma, but not of the normal lung epithelial cells adjacent to tumours [24,27]. In other reports, some positive staining of normal cells (albeit of lower intensity than with cancer cells) has been detected with two monoclonal anti-B1 antibodies [23,28,35]. In an exclusive immunohistochemical study using archived tissue sections of resected lung cancers in parallel with control non-cancer tissues from benign lung disease unrelated to cancer, B1 expression was also seen (at a frequency of 25%) in benign bronchial epithelial cells and inflammatory cells of the control group [28]. Nonetheless, hnRNP B1 expression is seen from the early stages of malignant transformation (in occult cancer, bronchial dysplasia and sputum) and is currently considered to be a more specific and sensitive biomarker than hnRNP A2/B1 for the early detection of lung cancer [24,25,27].

In the framework of the above discussion, we believe that our work brought out a number of important issues. A first observation concerns the cancer-related changes at the protein level within the highly related group of hnRNP A/B. Apart from the deregulation of hnRNP A2/B1 and B1 alone, there have been few reports on other hnRNP proteins. Our work focused on all three major hnRNP A/B-type proteins, namely hnRNP A2/B1, A1 and A3, whereas an additional hnRNP protein (hnRNP K) was included in some applications. The semi-quantitative estimates that were based on western blotting revealed the prominence of hnRNP A1 with respect to its higher degree of deregulation (range and frequency of over-expression) in NSCLC. By applying a threshold based on adjacent normal-appearing area separately in every patient, the tumour-to-normal ratio was found to be most significantly changed in the case of hnRNP A1 (76%), followed by hnRNP A3 (52%), A2/B1 (43%) and K/J (38%). (Table 2). The high frequency of hnRNP A1 over-expression in NSCLC seen in our study was a clear finding that confirmed and extended previous observations based on immunohistochemistry alone [30]. Over-expression of hnRNP A1 was not exclusive to NSCLC, as it was also seen in SCLC biopsies by immunohistochemistry (Valavanis C., personal communication). In addition, it did not appear to be restricted to lung cancer, as we had similar findings in breast cancer biopsies by immunoblotting (our unpublished observation). Increased expression of hnRNP A1 has also been reported in colon [37] and several other cancer types [17]. Our finding of hnRNP A3 over-expression is novel and requires further investigation for its potential application in lung carcinogenesis.

The alterations in the relative amounts of hnRNP A1 and A2/B1 we observed by western blotting were in overall agreement with the semi-quantitative estimates from the immunohistochemical analysis. Moreover, a clear distinction in the immunostaining pattern of hnRNP A1 and A2/B1 in the normal lung tissue was apparent. This had to do with a high threshold in the intensity of nuclear staining for hnRNP A2/B1 in the normal-appearing area of the lung (scoring index 1, 2 and few 3) in contrast to the very low staining index for A1 (Figure 3). This finding on A2/B1 staining intensity agrees well with previous observations. hnRNP A2/B1 protein and mRNA were expressed at almost equal levels in cancerous and non-cancerous tissues [16], and increased protein expression (around 40%) has been noted in both normal and abnormal bronchial epithelium of the lung in chronic smokers [33] and in non-neoplastic respiratory epithelium [20].

Moreover, immunohistochemical analysis showed mainly nuclear localisation of hnRNP A1 and A2/B1 in the cells within the cancer area, similar to the staining of the neighbouring normal-appearing cells. However, contradictory observations exist in the literature with respect to hnRNP cellular localisation in cancer cells. Both nuclear and cytoplasmic staining of hnRNP A2/B1 have been reported in [33,34]. Nuclear staining of hnRNP A2/B1 has also been observed in the case of mouse lung adenocarcinomas [38], and nuclear staining of the hnRNP B1 isoform has been reported by others [35]. However, the study of Pino et al. [30] on NSCLC tissues claimed primarily cytoplasmic localisation of hnRNP A2/B1, contrasting with the nuclear staining of hnRNP A1 and C1/C2, whereas hnRNP K was both nuclear and cytoplasmic. The cytoplasmic accumulation of hnRNP A2/B1 in association with tumour progression has been shown [21]. These discrepancies might be due to the different antibodies and fixation protocols applied.

The main objective of our study was the direct comparison of hnRNP A1 and A2/B1 at the protein and mRNA levels within the same patient. The high frequency (76%) of hnRNP A1 protein over-expression in cancer was not accompanied by similar changes in its steady-state mRNA level (15% up-regulation). In contrast to hnRNP A1, the same overall frequency (about 40%) of over-expression at the protein and mRNA levels was estimated for hnRNP A2/B1. Our study estimated the mRNA level of the B1 splicing variant alone, but it did not assess its protein level in parallel due to the unavailability in this framework of specific antibodies. However, the western blots using the anti-hnRNP A2/B1 antibody showed overall changes of B1 proportional to A2. Moreover, the frequency and range of B1 mRNA elevation in cancer were parallel to those of A2/B1 mRNA, although of lower significance (Table 3). Thus, our findings did not appear to agree with the report of Sueoka et al. [24] regarding elevated levels of hnRNP B1 mRNA compared to A2/B1 mRNA in lung cancer.

We also wish to comment that the lung cancer tissues used in our study were of a rather advanced cancer stage (low or intermediate differentiation). Nonetheless, the adjacent normal-appearing lung tissue 5 cm away from the boundary of the tumour area selected as a standard showed no signs of hyperplasia or metaplasia. Without doubt, a better control would have been the use of lung cancer biopsies from non-cancer patients, which would be expected to provide very low background levels of hnRNP A/B [16], but which were not available for the present study.

Our results provide strong evidence that supports the existence of distinct mechanisms responsible for hnRNP A/B deregulation in lung cancer, as suggested by a previous study [30]. This was manifested at the protein level by the frequent cases of opposing alterations (up- vs. down-regulation) in at least one of the hnRNP A/B members within the same biopsy. Most importantly, this apparent non-concurrent deregulation of hnRNP A/B protein levels was also reflected at the mRNA level. Based on the parallel quantification of the respective tumour-to-normal mRNA ratio, there was an absence of strict correlation between protein and mRNA levels in hnRNP A1. The high frequency (76%) of protein over-expression in cancer was not accompanied by similar changes in its steady-state mRNA level (15% up-regulation). We wish to note in this context that an apparently poor correlation between protein and mRNA levels in eukaryotic systems has been reported because quite often protein over-expression is not accompanied by similar changes at the mRNA level [39,40].

As noted in the Results, we detected in cancer tissue an alteration parallel to that of hnRNP A1 in both the protein and mRNA levels of ASF/SF2, a factor with a known antagonistic role to A1 in alternative splicing [12]. In a mouse model, although the relative amounts of both proteins changed during lung carcinogenesis, hnRNP A1 increased to a much higher extent in tumours (six-fold increase of hnRNP A1 relative to ASF/SF2) [14]. This also appears to be the case in human lung cancer, as in our study an increase of hnRNP A1 relative to ASF/SF2 was apparent in 80% of the cases (the majority with over a three-fold increase). These data add support to the existence of cancer-related alterations in the relative amounts of the splicing factors ASF/SF2 and hnRNP A1 that need to be further verified with respect to their significance in lung carcinogenesis.

Contradictory results on the usefulness of hnRNP A2/B1 for predicting early lung cancer still exist. Along these lines is a recent study by Zech et al. [29] on the diagnostic and prognostic value of hnRNP A2/B1 or B1 alone in lung cancer. By applying large-scale immunohistochemistry to frozen (not paraffin-embedded) sections of paired tumour/non-tumour biopsies from NSCLC patients, the frequency of A2/B1 over-expression in cancer was found to be particularly low; 10% compared to 91% of B1 in the cancer cells. In results contrasting with the report of Wu et al., [26] a prognostic value of B1 was not seen, whereas A2/B1 over-expression was associated with a negative prognosis [29]. As for the other discrepancies mentioned above, the existence of such contradictory results is thought to be related to the different antibodies and tissue fixation methods applied. In our study, we observed a higher frequency of hnRNP A1 over-expression than hnRNP A2/B1 over-expression, as seen both by western blotting and immunohistochemistry. The possibility nonetheless remains that hnRNP A1 alterations occur at a later stage of malignant transformation of the lung compared to A2/B1 or B1.

The present study has not addressed the question of the clinical usefulness of hnRNP A1 over-expression in the pathological diagnosis of lung cancer. However, the low score index for the staining of hnRNP A1 in the neighbouring non-involved lung tissue, when contrasted with the higher score of hnRNP A2/B1 in the same area (see Figure 3), highlighted the need for evaluating hnRNP A1 as a new molecular index that can be combined with the hnRNP B1 variant as well as other promising biomarkers (like telomerase expression and K-Ras mutations; discussed in [25]) for accurately predicting early lung carcinogenesis. Additionally, the observation of hnRNP A1 over-expression might provide a potential new target for disease management and intervention.

Currently, the molecular basis of deregulated expression of hnRNP proteins in lung cancer is not known. The involvement of signalling molecules that control hnRNP protein activity might be anticipated, as has been shown in the case of ASF/SF2 over-expression in breast and colon cancer [13]. A link between hnRNP A/B overexpression and carcinogenesis is provided by the finding on their requirement for cancer cell growth [17] and on the impairment of DNA repair upon hnRNP B1 over-expression [41]. Our study provided some initial indications related to the underlying mechanism(s) of altered hnRNP A/B expression in NSCLC. The differences seen in the pattern of deregulation between the two highly homologous proteins hnRNP A1 and A2/B1 are in line with the anticipated high complexity of the molecular events underlying malignant transformation in humans, with the intervention of mechanisms operating in a synergistic or an antagonistic way at several steps of the mRNA metabolism of a particular gene. One possible mechanism to explain hnRNP A1 protein over-expression in the absence of parallel changes in the corresponding mRNA level (shown in the present study) could involve translational repression of hnRNP A1 mRNA in non-tunourous normal cells that is lost in cancer cells and leads to protein accumulation. Such a mechanism may involve specific microRNA species that could target hnRNP A1 mRNA, in line with recent reports implicating the role of a number of microRNAs in lung carcinogenesis [42]. Whether such a mechanism exists in the case of hnRNP A1 mRNA remains to be shown. Nonetheless, caution should be taken when interpreting results based on gene expression analysis at the mRNA level alone without considering changes in the cell's proteome. Relevant future studies aiming to define the specific mRNA subsets associated with lung carcinogenesis that are most affected by hnRNP A/B over-expression would be of particular interest.

## Conclusion

In this study, we demonstrate altered expression of the splicing factors hnRNP A/B and ASF/SF2 in cancer patients with non-small cell lung cancer. The application of semi-quantitative protein estimates and real time RT-PCR analysis on paired cancer/non-cancer lung biopsies from 21 patients allowed for direct comparisons of the protein and corresponding mRNA expression profiles to be made. We provide evidence for mostly uncoupled deregulation among members of the hnRNP A/B group (A1, A2/B1 and A3). hnRNP A1 was the protein species with the highest degree and frequency of altered expression, but it also showed a clear lack of correlation between its protein and mRNA expression levels. These findings add to our current understanding of hnRNP A/B deregulation in lung cancer, and further studies evaluating their potential application as diagnostic/prognostic biological markers are warranted.

## Competing interests

The authors declare that they have no competing interests.

## Authors' contributions

GB carried out the study of protein and mRNA levels, performed statistical analysis and participated in drafting the manuscript. MP-G assisted in tissue handling, data interpretation and manuscript preparation. CV provided the material and together with ML carried out tissue pathology, clinical characteristics and immunohistochemical analysis of tissue sections. AG participated in the design of the study, coordination and manuscript composition. All authors read and approved the final manuscript.

## Pre-publication history

The pre-publication history for this paper can be accessed here:

http://www.biomedcentral.com/1471-2407/10/434/prepub
